# Protective Efficacy and Immunogenicity of a Combinatory DNA Vaccine against Influenza A Virus and the Respiratory Syncytial Virus

**DOI:** 10.1371/journal.pone.0072217

**Published:** 2013-08-14

**Authors:** Viktoria Stab, Sandra Nitsche, Thomas Niezold, Michael Storcksdieck genannt Bonsmann, Andrea Wiechers, Bettina Tippler, Drew Hannaman, Christina Ehrhardt, Klaus Überla, Thomas Grunwald, Matthias Tenbusch

**Affiliations:** 1 Department of Molecular and Medical Virology, Ruhr-University Bochum, Bochum, Germany; 2 Ichor Medical Systems, San Diego, California, United States of America; 3 Institute of Molecular Virology, Centre of Molecular Biology of Inflammation, Westfaelische Wilhelms University, Muenster, Germany; University of Georgia, United States of America

## Abstract

The Respiratory Syncytial Virus (RSV) and Influenza A Virus (IAV) are both two major causative agents of severe respiratory tract infections in humans leading to hospitalization and thousands of deaths each year. In this study, we evaluated the immunogenicity and efficacy of a combinatory DNA vaccine in comparison to the single component vaccines against both diseases in a mouse model. Intramuscular electroporation with plasmids expressing the hemagglutinin (HA) of IAV and the F protein of RSV induced strong humoral immune responses regardless if they were delivered in combination or alone. In consequence, high neutralizing antibody titers were detected, which conferred protection against a lethal challenge with IAV. Furthermore, the viral load in the lungs after a RSV infection could be dramatically reduced in vaccinated mice. Concurrently, substantial amounts of antigen-specific, polyfunctional CD8^+^ T-cells were measured after vaccination. Interestingly, the cellular response to the hemagglutinin was significantly reduced in the presence of the RSV-F encoding plasmid, but not vice versa. Although these results indicate a suppressive effect of the RSV-F protein, the protective efficacy of the combinatory vaccine was comparable to the efficacy of both single-component vaccines. In conclusion, the novel combinatory vaccine against RSV and IAV may have great potential to reduce the rate of severe respiratory tract infections in humans without increasing the number of necessary vaccinations.

## Introduction

Influenza A Virus and the Respiratory Syncytial Virus are causative agents of severe respiratory tract infection especially in young children and elderly people. The global disease burden is estimated to ∼600 million and ∼60 million cases per year for IAV and RSV, respectively, leading to estimated ∼0.5 million deaths/year worldwide (www.who.int). Vaccinations against both viruses would provide therefore a substantial cost reduction in the global health system, as demonstrated by the already licensed vaccines against seasonal IAV [1]. Nevertheless, the production processes of these vaccines (e.g. subunit vaccine, whole inactivated virus vaccine) are very time-consuming and the efficacy is modest and short-lived. Thus, alternative strategies to reduce the production timeline and increase the efficacy are highly appreciable. In addition, there is no prophylactic vaccine against the RSV available so far. Recently, DNA vaccines have demonstrated great potential as an alternative vaccine platform capable of inducing protective immune responses against a variety of infectious diseases in preclinical models (reviewed in [2]), including RSV [3], [4] and IAV [5]. The implementation of more effective delivery methods, like electroporation, and the use of codon-optimized expression systems had further boosted the immunogenicity of such vaccines. DNA vaccines for a wide range of disease indications have advanced into human clinical trials and several are approved for use in the field of veterinary medicine (reviewed in [6], [7]). In our previous work, we successfully generated DNA vaccines providing protection against RSV or IAV using expression plasmids encoding the viral surface proteins RSV-F or the hemagglutinin of IAV, respectively [3], [5]. Given the overlap in populations vulnerable to these respiratory infections, assessing the feasibility of combining these DNA vaccines represents a logical strategy. Such combinatory vaccines could reduce the number of immunizations an individual needs, leading to enhanced compliance and improved cost effectiveness. This concept was successfully established for many pediatric vaccines, like the mumps-measles-rubella (MMR) vaccine. Nevertheless, there are also reports on reduced immunogenicity or efficacy of conventionally developed combinatory vaccines in comparison to the respective single-component vaccines, e.g. Hepatitis A and B vaccine [8]. For DNA vaccines, it has already been demonstrated that plasmids encoding different antigens from either the same [9], [10] or a second pathogen could induce substantial immune responses against both antigens [11], [12]. However, in other studies, the simple addition of two expression plasmids encoding NP and M2 from IAV reduced the protective capacity of a combinatory DNA vaccine encoding HA and NA from the same virus by 30% [13]. This is not unexpected due to immunological interference within the recipient, thus indicating the need for an extensive immunogenicity analysis for each combinatory DNA vaccine. The mechanisms of such immunological interference are not yet fully understood, but possible explanations include interference in the antigen presenting pathway and/or alterations at the level of transcription/translation leading to changes in antigen expression levels. In the present study, we tested the immunogenicity of a combinatory DNA vaccine encoding the viral surface proteins HA from IAV (IAV-HA) and the F protein from RSV (RSV-F). The vaccines were delivered by intramuscular electroporation and the cellular as well as humoral immune responses to both antigens were analyzed in depth to rule out any form of immunological interference. Furthermore the protective efficacy of the combinatory vaccine was compared to the ones of the single component vaccines.

## Materials and Methods

### Plasmids and Vaccines

The plasmid pF^syn^, based on pcDNA3.1, contained the codon-optimized sequence of the full-length RSV-F protein and is described elsewhere [14]. The codon-optimized sequence of the HA of the virus strain A/Puerto Rico/8/34 was synthesized by Geneart (Regensburg, Germany), followed by PCR amplification and cloning into the pVAX backbone. The HA sequence is followed by an ollas-tag [15] for protein detection and the resulting plasmid is referred to as pHA^syn^. A non-coding pcDNA3.1 plasmid was used as control plasmid. DNA for immunization was prepared using the NucleoBond® Xtra Maxi EF Kit (Macherey-Nagel, Düren, Germany) and tested for endotoxin levels with the LAL quantification assay (Cambrex Bio Science, Verviers, Belgium), confirming that the dose used for immunization of mice contained less than 0.1 endotoxin units (EU).

### Expression Analysis

293T cells were transiently transfected using PEI (polyethyleneimine) with either 1 µg or 10 µg of plasmid DNA as described elsewhere [16]. The two plasmids pHA^syn^ and pF^syn^ were transfected either in combination with each other or with pcDNA3.1. The influence on the expression levels of both viral proteins were analyzed by Western Blot analyses. Cell lysates were prepared 48 h after transfection. The protocol for the detection of RSV-F by Western blot under non-reducing conditions is described in a previous study [14]. For the detection of IAV-HA, a monoclonal anti-HA antibody was purified from hybridoma culture and used together with an HRP-conjugated anti-mouse IgG (DakoCytomation, Glostrup, Denmark) antibody in Western blots under reducing conditions, as described elsewhere [17]. The housekeeping protein α-tubulin was used as loading control and detected by a polyclonal antibody (Rockland, Gilbertsville, PA, USA) in combination with an HRP-conjugated anti-rabbit antibody (DakoCytomation, Glostrup, Denmark). A semi-quantitative, densitometry analysis was performed with the software Wasabi! (Hamamatsu Photonics Germany GmbH, Herrsching am Ammersee, Germany).

### Animals and Immunizations

6–8 week old female BALB/cJRj mice were purchased from Janvier (Le Genest-ST-Isle, France) and housed in individually-ventilated cages in accordance with the national law and institutional guidelines. The study was approved by an external ethics committee authorized by the North Rhine-Westphalia State Office for Consumer Protection and Food Safety and performed with the project licenses (AZ 87-51.04.2010.A302). The injection and electroporation procedure was performed in accordance to the manual supplied by the manufacturer (Ichor Medical Inc., San Diego, USA) and is described elsewhere [5], [18]. The respective amounts of the DNAs (Table 1) were diluted in 100 µl PBS and the animals were immunized twice within a 3-week interval under ketamine (50 mg/kg)/xylazine (10 mg/kg) anesthesia. One week after the second vaccination, the animals were sacrificed by cervical dislocation and the cellular immune responses were analyzed by intracellular cytokine staining of isolated splenocytes. Mice were bled under general anesthesia by exposing them briefly to isofluran (Paragos, Frankfurt, Germany) vapors, and then 15 drops of blood were drawn from the retro-orbital sinus using a glass capillary tube. Blood samples were collected on days 20 and 49 and analyzed for antigen-specific antibodies. The protective efficacies of the vaccines were determined by a challenge infection with either RSV or IAV five weeks after the final immunization.

**Table 1:** Experimental setup.

Group	Vaccine (d0+d21)	IAV-Challenge (d56)	RSV-Challenge (d56)
**Naïve**	–	+	+
**(HA)^low^**	2 µg pHA^syn^ +2 µg pcDNA3.1	+	–
**(F)^low^**	2 µg pF^syn^ +2 µg pcDNA3.1	–	+
**(HA+F)^low^**	2 µg pHA^syn^ +2 µg pF^syn^	+	+
**(HA)^hi^**	20 µg pHA^syn^ +20 µg pcDNA3.1	+	+
**(F)^hi^**	20 µg pF^syn^ +20 µg pcDNA3.1	+	+
**(HA+F)^hi^**	20 µg pHA^syn^ +20 µg pF^syn^	+	+

### Intracellular Cytokine Staining

One week after the second immunization spleen lymphocytes were isolated and re-stimulated as previously described [5]. Briefly, CD8^+^ T-cells were re-stimulated for 6 h in the presence of monensin (2 µM), αCD107a-FITC (1 µl, BD Bioscience, Germany) and the peptide HA_532–540_ (IYSTVASSL), 5 µg/ml) or the combined RSV-F peptides (DKYKNAVTELQLLMQ; VTTPVSTYMLTNSEL; VSTYMLTNSELLSLI, each 5 µg/ml). Non-stimulated splenocytes were used as controls for unspecific cytokine production. After the stimulation, surface staining was performed with αCD8-PerCP or αCD4-PerCP antibodies (BD Bioscience, Heidelberg, Germany). Cells were fixed in 2% paraformaldehyde and permeabilized with 0.5% Saponin in PBS/BSA/azide buffer. Cytokines were detected with αIFN-γ-PE and αIL-2-APC on a FACSCalibur® flow cytometer (BD Bioscience, Germany).

### Serological Assays

To analyze the sera for antibodies recognizing the viral antigens in their membrane-bound conformation, a flow cytometric analysis was performed with transfected 293T cells as previously described [5]. Briefly, 293T cells were transfected with the IAV-HA or RSV-F expressing plasmid two days prior incubation with the sera of vaccinated animals. Antibodies bound to the surface proteins of the transfected cells were detected via FITC-labeled anti mouse IgG antibodies (BD Bioscience, Germany). The mean fluorescence intensities of the cells correspond to the level of antigen-specific antibodies in the sera.

To quantify IAV and RSV specific IgG1 and IgG2a antibody levels, ELISA were performed as previously described [19]. Briefly, 96-well plates were coated overnight at 4°C with heat-inactivated Influenza A/PR/8/34 or RSV A/Long (10^6^ plaque forming units (PFU)/well). The sera were diluted 1–100 with PBS containing 2% fat dried milk powder and 0.5% Tween20 and incubated for 1 h. Finally, horseradish peroxidase-coupled antibodies against mouse IgG1 or IgG2a antibodies (BD Bioscience) were used for the detection.

To determine influenza specific neutralizing antibody titers, mouse sera were 2-fold serially diluted in 100 µl DMEM (Invitrogen) containing 0.6% BSA, 1% penicillin-streptomycin and 0.12% trypsin and pre-incubated with 2000 PFU of Influenza A/PR/8/34 at 37°C for 1 h. In the next step, the serum-virus mix was applied to MDCK cells, which had been seeded at 5×10^4^ cells/well the day before, and incubated at 37°C for 1.5 h. Finally, additional 150 µl of medium were added to the cells. After 96 h incubation at 37°C non-infected wells were identified by staining with crystal violet dye. The reciprocal dilution of the sera completely inhibiting infection was considered as neutralizing antibody titer.

RSV specific neutralizing antibody assays were performed using rgRSV in accordance with the protocol described previously [3].

### Challenge Experiments

5–6 weeks after the second immunization mice were anesthetized with ketamine/xylazine and challenged intranasally with either influenza A/PR/8/34 (250 PFU) or RSV A/Long (10^6^ PFU) diluted in 50 µl PBS [3]. The weight loss of the mice was monitored daily after the infection as an indicator of disease progression. Mice were sacrificed six (Influenza) or five (RSV) days after infection and the lungs were removed for the quantification of the viral load. Lungs were homogenized in 2 ml PBS using the GentleMACS Dissociator (Milteny) according to the manufacturer’s protocol. Viral RNA was isolated from lung homogenates using the QIAamp® Viral RNA Mini Kit (Qiagen) according to the manufacturer’s instructions. The quantity of viral RNA was determined by reverse transcription real time PCR (RT-qPCR) using the QuantiTect™ Probe RT-PCR Kit (Qiagen) with SYBR-Green. The following specific primers were used: Influenza (*cttctaaccgaggtcgaaacg*+*agggcattttggacaaag/tcgtcta)* [20]; RSV (*agatcaacttctgtcatccagcaa*+*gcacatcataattaggagtatcaat*) [3]. The detection limits were 5 copies/PCR for the Influenza-RT-PCR and 50 copies/PCR for the RSV-RT-PCR, which corresponds to 428 and 4280 copies/ml lung homogenate, respectively.

### Statistical Analysis

Results are expressed as the means ± standard errors of the means (SEM). Statistical comparisons were performed by one-way ANOVA test, followed by a Tukey post test using the Prism 5.0, GraphPad Software. A P value of <0.05 was considered to be statistically significant.

## Results

### Influence of Co-delivery of DNA Vaccines on Antigen Expression Levels

**Figure 1:**
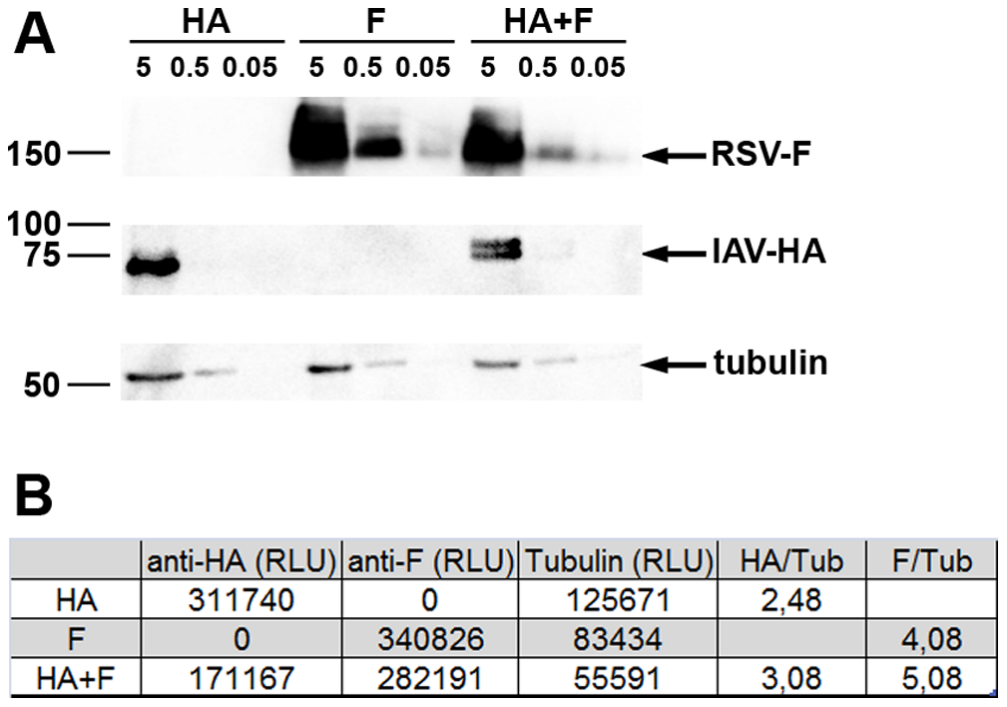
Expression analysis. 293T cells were transiently transfected with the IAV-HA or RSV-F expressing plasmids either alone or in combination. To guarantee same amounts of input DNA, pcDNA3.1 was added to the transfection samples of a single plasmid. The transfections were done with 0.05, 0.5 or 5 µg per plasmid and cell lysates were prepared 48 h after transfection. The expression of the viral surface proteins was detected by Western blot analyses under either denaturing conditions for the detection with an anti-HA antibody or non-reducing conditions for the detection with an anti-RSV-F antibody. The detection of α-tubulin served as a loading control (**A**). A semi-quantitative, densitometry analysis was performed with the software Wasabi! for the lysates obtained after transfecting 5 µg of DNA. Listed are the RLUs detected in the different Western blots (**B**).

In this study, we compared the efficacy of a combinatory DNA vaccine to the efficacy of single component vaccines. Therefore we had to confirm that the simultaneous delivery of both plasmids did not result in a considerable loss of expression of either antigen. 293T cells were transfected with the IAV-HA and RSV-F encoding plasmids either together with each other or with an empty control plasmid (pcDNA3.1). Cells were transfected with 0.05, 0.5 or 5 µg per plasmid and lysed two days later. The proteins were detected by Western blot analyses using HA-, RSV-F- or α-tubulin-specific antibodies and all proteins appeared at the expected size. (Fig. 1A). A semi-quantitative, densitometry analysis revealed that the expression of neither HA nor RSV-F was significantly inhibited by the presence of the second expression plasmid (Fig. 1B). Although the total amount of HA and RSV-F were slightly lower in the presence of the second antigen encoding plasmid than in the presence of the control plasmid, the ratios to the internal control protein, α-tubulin, were not affected. This indicates that there is no intracellular inhibition by one of these viral proteins on the expression rate of the other one *in vitro.*

### Cellular Immune Response after Combinatory Immunization

**Figure 2:**
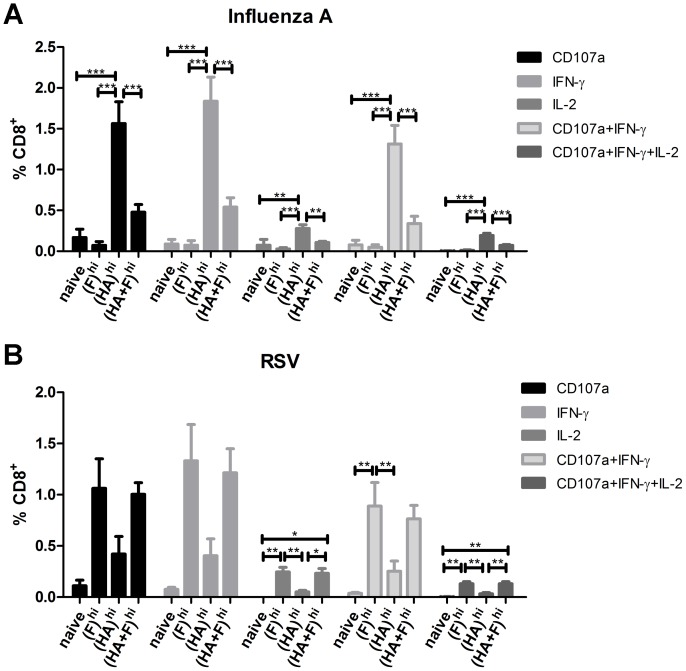
Antigen-specific CD8^+^ T-cell responses. Balb/c mice were immunized according to Table 1. Since the low dose regimen did not result in substantial responses, only the groups which received a total of 40 µg of plasmid DNA were included. IAV-HA (**A**)- and RSV-F (**B**)-specific CD8^+^ T-cell responses were analyzed one week after the second immunization by staining for the degranulation marker CD107a and intracellular staining for the inflammatory cytokines IFN-γ and IL-2. The percentages of the different populations among the total CD8^+^ T-cells are shown. Mean values and standard errors of the means (SEM) represent 8 mice per vaccine group out of two independent experiments and 4 mice for the naïve group. (*** = p<0.001, ** = p<0.01, * = p<0.05; 1 way-ANOVA, Tukey post-test).

To compare the immunogenicity of the combinatory vaccine to that of the single component vaccines, mice were immunized twice with different doses of DNA (hi =  20 µg; low =  2 µg) or left untreated according to Table 1. The single component vaccines were mixed with an empty pcDNA-vector to keep the total amount of plasmid constant. Additionally, the high-dose (20 µg) of the irrelevant antigen-expressing plasmid were always used as a control for unspecific immune stimulation by the vaccination procedure itself; i.e. (HA)^hi^ serves as a control for all RSV-specifc assays and (F)^hi^ for the IAV-specific ones. The cellular immune responses were analyzed one week after the second immunization by intracellular cytokine staining (Fig. 2). Since two immunizations with low doses of the plasmids (2 µg each) did not result in substantial responses, only the results of the groups, which received high doses of the antigen-expressing plasmid were shown. In accordance with our previous studies, 20 µg of the IAV-HA expressing plasmid, group (HA)^hi^, induced high numbers of antigen-specific CD8^+^ T-cells (Fig. 2A), mainly characterized by the expression of CD107a and IFN-γ (ca. 1.3% of all CD8^+^ T-cells). Surprisingly, the immune response was significantly reduced (∼0.5%) if the RSV-F encoding plasmid was co-applied (HA+F)^hi^. This could be observed for all subpopulations of antigen-specific CD8^+^ T-cells analyzed in this ICS assay (Fig. 2A). Furthermore, the HA-specific CD4^+^ T-cell responses detected by intracellular cytokine staining for the T_H_1 cytokines IFN-α, TNF and IL-2 were also reduced in the presence of the RSV-F encoding plasmid (Fig. S1A). We further measured the typical T_H_2 cytokines IL-4, IL-5, IL-10 and IL-13 in the supernatant of HA-stimulated splenocytes and could not detect any IL-5 or IL-10 production. Interestingly, the IL-4 production, although at a low level, was comparable for (HA+F)^hi^ and (HA)^hi^, whereas the IL-13 production was again significantly reduced in the combined vaccine group (Fig. S1B).

In contrast to the IAV-HA-specific response, the RSV-F-specific CD8^+^ T-cell response was not significantly different between the groups (HA+F)^hi^ and (F)^hi^ (Fig. 2B). In both groups nearly 1% of all CD8^+^ T-cells were positive for CD107a and IFN-γ after RSV-F peptide stimulation. Unexpectedly, in the control group treated with the IAV-HA expressing plasmid (HA)^hi^, some T-cells also reacted specifically to the RSV-F peptides used in the assay, indicating possibly cross-reactive epitopes. Unfortunately, this led to a high variation of reacting CD8^+^ T-cells in these animals so that the multiple group comparison by 1-way ANOVA did not reach statistical significance for some subpopulation (e.g. IFN-γ+/CD8+ cells: naïve vs. F^hi^). However, in all populations analyzed no reduction of the RSV-F-specific response could be observed in the presence of the IAV-HA expressing plasmid.

### Humoral Immune Response after Combinational Immunization

**Figure 3:**
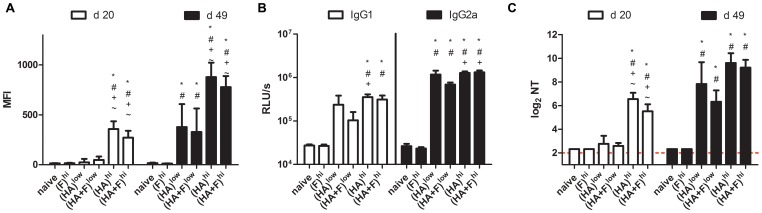
Influenza-specific antibody response. Balb/c mice were immunized according to Table 1. Sera were collected at days 20 and 49 and IAV-HA-specific antibody responses were analyzed. To analyze antibodies binding IAV-HA in its membrane bound conformation, the sera were incubated with IAV-HA expressing 293T cells and bound antibodies were subsequently detected by FITC-labeled anti-mouse IgG antibodies. The mean fluorescence intensities (MFI) of each group (means+SEM) are shown for sera from day 20 and day 49 (**A**). The distribution of IAV-HA-specific IgG1 and IgG2a were analyzed in an ELISA using IAV coated plates and HRP-conjugated anti-IgG1 and IgG2a antibodies. The means and SEM of the relative light units (RLU) are shown for sera from day 49 (**B**). The neutralizing antibody titer (NT) was analyzed by a microneutralization assay. The reciprocal value of the serum dilution which results in complete protection from infection is given as neutralizing titer. The mean and SEM of each group are indicated for the sera of day 20 and day 49 (**C**). The results for each group represent at least 12 mice out of 2–3 independent experiments with the exception from the results of group (HA)^low^, which based on a single experiment with 6 mice. (p<0.05): * vs. naïve; **^#^** vs. (F)^hi^; **^+^**vs. (HA+F)^low^; ^∼^ vs. (HA)^low^ (1 way-ANOVA, Tukey post-test).

**Figure 4:**
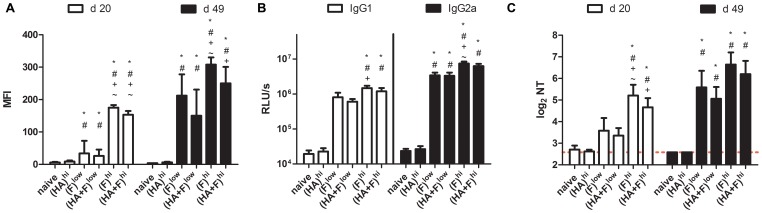
RSV-specific antibody response. Balb/c mice were immunized according to Table 1. Sera were collected at days 20 and 49 and RSV-F-specific antibody responses were analyzed. To analyze antibodies binding RSV-F in its membrane bound conformation, the sera were incubated with RSV-F expressing 293T cells and bound antibodies were subsequently detected by FITC-labeled anti-mouse IgG antibodies. The mean fluorescence intensities (MFI) of each group (means+SEM) are shown for sera from day 20 and day 49 (**A**). The distribution of RSV-F-specific IgG1 and IgG2a were analyzed in an ELISA using RSV coated plates and HRP-conjugated anti-IgG1 and IgG2a antibodies. The means and SEM of the relative light units (RLU) are shown for sera from day 49 (**B**). The reciprocal value of the serum dilution which results in 50% inhibition of infection with rgRSV is given as neutralizing antibody titer (NT). The mean and SEM of each group are indicated for the sera of day 20 and day 49 (**C**). The results for each group represent at least 12 mice out of 2–3 independent experiments. (p<0.05): * vs. naïve; **^#^** vs. (HA)^hi^; **^+^**vs. (HA+F)^low^; ^∼^ vs. (F)^low^ (1 way-ANOVA, Tukey post-test).

Since virus-specific antibodies are important for protection against the infection with these two viruses, we analyzed the antibodies in the sera of the vaccinated mice in regard to their binding capacity to the natural membrane-bound surface protein, to their IgG-subtype and their neutralization efficacy *in vitro* (Fig. 3 and 4). Three weeks (day 20) after the first immunization, IAV-HA-specific antibodies could be detected in the groups (HA)^hi^ and (HA+F)^hi^, but not in the low-dose groups. These antibodies were able to bind the HA protein expressed on the surface of transfected cells and could neutralize the A/PR/8/34 virus *in vitro* at serum dilutions ranging from 1/10 to 1/640 (Fig. 3A and C). These antibody responses were substantially boosted by the second immunization and the mean neutralizing antibody titers increased to 775 and 592 for (HA)^hi^ and (HA+F)^hi^, respectively. After the second immunization, the animals of the low dose groups (HA)^low^ and (HA+F)^low^ also had neutralizing antibody titers significantly elevated above control animals (Fig. 3C). Although the antibody response measured by the FACS-based assay were still significantly lower in these two groups compared to that in the high dose groups (Fig. 3A), the differences did not reach statistical significance in the micro-neutralization assay (Fig. 3C). To further characterize the IgG subtype of the virus-specific antibodies, virus-coated ELISA plates were incubated with the sera of day 49 after first immunization and bound antibodies were detected by IgG1- and IgG2a-specific antibodies (Fig. 3B). All immunized animals showed a balanced IgG1/IgG2a response with no significant differences between the groups, which received one or two antigen expressing plasmids. Overall the IAV-HA-specific humoral immune response was comparable for animals treated with the combinatory or the single-component vaccine. The same kind of analyses were performed to detect RSV-F-specific antibodies and revealed a very similar picture (Fig. 4). Again, the high vaccine dose induced neutralizing antibodies as early as twenty days after the first immunization, whereas the animals of the groups (F)^low^ and the (HA+F)^low^ needed a second immunization to produce comparable levels of RSV-neutralizing antibodies (Fig. 4C). Nevertheless, the levels of serum antibodies binding RSV-F on the surface of transfected cells were significantly higher at day 49 in the high dose groups (F)^hi^ and (HA+F)^hi^ than in the respective low dose groups (F)^low^ and (HA+F)^low^ (Fig. 4A). Similar to the IAV-HA-specific response, the distribution of RSV-specific antibodies is balanced for the subtypes IgG1 and IgG2a (Fig. 4B). Overall, the combinatory vaccine induced antibody responses comparable to the single-component vaccines, including high amounts of binding and neutralizing antibodies specific for both viral surface proteins. Furthermore, no evidence of anti-DNA antibodies were detected in the sera of vaccinated mice (data not shown).

### Protective Efficacy Against RSV and IAV Infections

**Figure 5:**
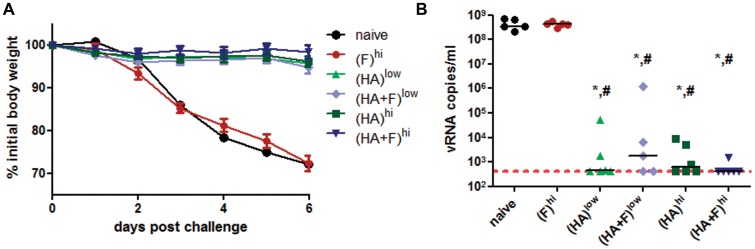
Protection from Influenza A Virus challenge. Balb/c mice were immunized according to Table 1. Five weeks after the second immunization, the mice were challenged with 250 PFU IAV (PR/8/34). The weight loss of each animal was monitored daily and the means and SEM (n = 5–6) are indicated for each group up to day 6 post challenge (**A**). At day 6, viral loads in lung homogenates (vRNA copies/ml) were measured by qRT-PCR. Each animal is represented by one dot and the means of each group are marked by the line. The dotted line indicates the detection limit (428 copies/ml) (**B**). (p<0.05): * vs. naïve; **^#^** vs. (F)^hi^ (1 way-ANOVA, Tukey post-test).

Following confirmation of vaccine induced anti-RSV and anti-IAV immune responses, the protective efficacy was assessed by experimental infections via the intranasal route. In accordance with the high neutralizing antibody titers against IAV, all vaccinated animals were fully protected against disease progression indicated by constant weights after IAV challenge infection (Fig. 5A). In contrast, all animals of the two control groups, naïve and (F)^hi^, started to lose weight two days post infection and had to be sacrificed by day 6 due to loss of more than 25% of the initial body weight. To further analyze the protective efficacy achieved by vaccination, the viral loads were measured in the lung homogenates at day 6 post infection (Fig. 5B). There is no evidence of non-specific protection conferred by the application of the DNA electroporation as demonstrated by the animals of group (F)^hi^, which had comparable viral loads as the naïve control animals. The median viral load was reduced at least by 5 logs for all groups, which received the IAV-HA-expressing plasmid (Fig. 5B). The best protection was observed in the group (HA+F)^hi^, in which the viral loads of five out of six animals were below the detection limit of the qRT-PCR and therefore not distinguishable from non-infected controls. Nevertheless, there were neither statistical significant differences in the efficacy between the low and the high dose regimen nor between the single-component and the combinatory vaccines.

**Figure 6:**
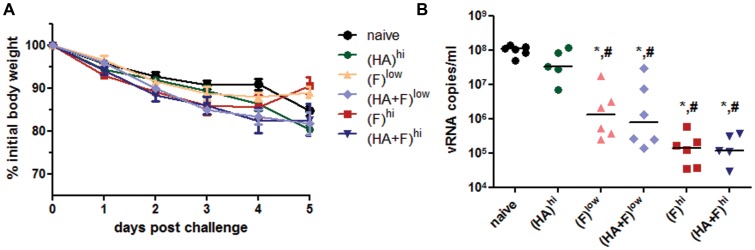
Protection from RSV challenge. Balb/c mice were immunized according to Table 1. Five weeks after the second immunization, the mice were challenged with 10^6^ PFU RSV (A/Long). The weight loss of each animal was monitored daily and the means and SEM (n = 5–6) are indicated for each group up to day 5 post challenge (**A**). At day 5, viral loads in lung homogenates (vRNA copies/ml) were measured by qRT-PCR. Each animal is represented by one dot and the means of each group are marked by the line. The detection limit of the qRT-PCR was 4280 copies/ml (**B**). (p<0.05): * vs. naïve; **^#^** vs. (HA)^hi^ (1 way-ANOVA, Tukey post-test).

In contrast to the IAV infection, the infection with the RSV is not lethal in mice and there is only a minor weight loss detectable in non-vaccinated mice. Although there are some variations in the weight loss from animal to animal, there were no significant differences between the groups at any time point (Fig. 6A). In contrast, the viral loads in the lung were statistically significant reduced in all animals which received the RSV-F expression plasmid compared to the naïve animals (Fig. 6B). Interestingly, the animals of group (HA)^hi^ had also 3-fold lower copy numbers of viral RNA than the naïve animals. This is in line with the observation that in some animals of this group CD8^+^ T-cells reactive to the RSV-F specific peptides were detected (Fig. 2B). Again, there were no differences in the efficacy of the single-component, (F)^hi^ and (F)^low^, and the combinatory vaccines, (HA+F)^hi^ and (HA+F)^low^. The median viral load were reduced in the low dose groups by a factor of 83 for (F)^low^ and 136 for (HA+F)^low^. Although it did not reach statistical significance in the multi-group comparison, the viral load could be further reduced by 1-log if the higher dose of the expression plasmid was used (Fig. 6B).

We could demonstrate that the combinatory DNA vaccine protected at least as efficient as the single-component vaccine against infections of both viruses. Furthermore, the protection in both models strongly correlated with the neutralizing antibody titer (data not shown).

## Discussion

In the present study, we thoroughly compared the immunogenicity and efficacy of a combinatory DNA vaccine against IAV and RSV to those of the single vaccines. Although Talaat *et al*. already reported on a protective combinatory DNA vaccine comprising of expression plasmids for HA (IAV), F (RSV) and gD (HSV) [11], our study was different in regard to the application via electroporation and to codon-optimized sequences for both antigens, which dramatically increased the immunogenicity and efficacy of our DNA vaccines. Furthermore, we analyzed the cellular and humoral immune responses to both antigens in detail to detect any immunological interference between the two viral surface proteins. One of the most impressive advantages of gene-based vaccines is the induction of highly-efficient CTL responses in addition to the antibody response. Interestingly, co-administration of an RSV-F expressing plasmid reduces the CD8^+^ and CD4^+^ T-cell responses to IAV-HA, but the CD8^+^ T-cell response to RSV-F was not influenced by the addition of the IAV-HA expressing plasmid compared to the single plasmid vaccination. This is in contrast to the work of Patel *et al.*, in which the co-administration of an IAV-HA expressing plasmid of an H5 subtype led to reduced cellular responses to NA and NP, encoded by the second plasmid [13]. These observations suggest a kind of immunological hierarchy, which strongly depends on the chosen antigens and cannot be predicted for other combinations. This is in line with previously published reports on immunodominance during viral infections and that subdominant epitopes can become more immunogenic if the immunodominant was deleted [21]–[23]. An alternative explanation for the reduced T-cell responses against the IAV-HA might be a direct effect of immune-modulatory properties of the F protein. Since RSV-F was reported to be an TLR-4 agonist [24] and that TLR-4 signaling can trigger T_H_2 responses [25], [26], it might have been possible that the HA-specific CD4 response was shifted from a T_H_1 to a T_H_2 response in the presence of RSV-F. But we could not detect a significant increase in IL-4, IL-5 or IL-10 production after HA-specific stimulation. In contrast, the IL-13 production was reduced in the same manner as it was observed for the IFN-γ production by HA-specific T-cells. This rather suggests a more general immunosuppression by RSV-F than a T_H_2 bias. In this context, it was reported that the interaction with RSV-F could inhibit the proliferation of peripheral blood lymphocytes *in vitro* [27], which might also result in lower T-cell priming *in vivo*. Since the HA used in this study is derived from the PR8/34 virus and is usually well expressed, the immunosuppressive effect of RSV-F might be even more prominent for HA variants with lower expression levels. This might be partially circumvented by using codon-optimized sequences, which could overcome instability issues of the RNA sequences [5], but not in cases where the amino acid sequence itself influences the expression level.

The high dose of the combinatory DNA vaccine induced substantial, polyfunctional CD8^+^ T-cell responses to both antigens, which should rapidly control viral replication once infection takes place. This might be of particular interest, since it was shown that CD4^+^ and CD8^+^ T-cell responses to influenza could partially confer protection from heterologous IAV infection [28]–[31]. It is further known that CTL responses play an important role in controlling RSV infection [32]–[34]. Interestingly, in some of the mice which received only the HA encoding plasmid, CD8^+^ T-cells produced IFN-γ after RSV-F-specific re-stimulation indicating some possible cross-reactivity which needs further evaluation. Consistently, the viral load in this group was 3-fold lower than in naïve animals which might be due to cytotoxic T-cells. Since there is no direct overlap in the RSV-F- and HA-specific peptides we used, it might be possible that some similarities in the amino acids at anchor positions might account for this cross-reactivity of the CD8^+^ T-cells as it was reported for HIV-specific T-cells [35].

Since the inactivated influenza virus vaccines approved for use in humans are known to be poor inducers of cellular immune responses, it is commonly accepted that protection from IAV infection is mainly based on antibody mediated mechanisms, like neutralization of the virus. We therefore analyzed carefully the humoral immune responses induced either by the combinatory vaccine or the single components. Although we found significant differences in the CD4^+^ T-cell responses to IAV-HA, the antibody response to IAV-HA seemed to be comparable for both groups. This was true in regard to the antibodies binding to membrane-anchored protein, to the neutralizing capacity and also to the IgG1/IgG2a distribution. Additionally our experiments demonstrate that two immunizations even with a low dose of 2 µg per plasmid were sufficient to provide protective antibody responses. This confirms the finding of Zhou *et al.*, who used a comparable vaccination protocol with either 5 µg or 30 µg of an H5 expressing plasmid and could show that the higher dose induced strong antibody responses already after the initial priming, whereas a second shot was needed for the lower dose [36]. In our previous work, we found the neutralizing antibody titers induced by this vaccination protocol to be comparable to those in humans after a single vaccination with a commercial H1N1(2009) vaccine (Pandemrix©) ([5] and unpublished data).

The humoral immune response to RSV-F seemed not to be influenced by the presence of the second antigen as well, which was also described in the previous study by Talaat *et al*. [11]. Nevertheless, the application via electroporation strikingly enhanced the antibody response compared to our previous study using conventional subcutaneous injections [3] and was nearly equal in strength to recombinant adenoviral vector immunizations [37], underlining the great potential of DNA electroporation. Since it is still of debate that a dominant T_H_2 response might aggravate the course of a subsequent RSV infection [38], [39], it is important to note, that our DNA vaccine induced a balanced T_H_1/T_H_2 immune response and that there was no sign of induction of an enhanced disease after challenge.

We could demonstrate that the efficacy of the combinatory DNA vaccines was at least as high as the ones of the single-component vaccines. This was again in line with the previous report of Talaat *et al.*, in which the efficacy was not reduced by mixing IAV-HA, RSV-F and HSV-gD expressing plasmids. Nevertheless, we were able to reduce the viral load of RSV after infection by nearly 1000-fold, which was much more efficient than the 10-fold reduction described for the earlier combinatory DNA vaccine [11] and in the range of protection observed after the recombinant adenoviral vector immunization [37]. Furthermore, the vaccinated mice were fully protected against disease progression after an IAV infection and in half of them the viral load in the BALs and the lungs were even below the detection limit of the qRT-PCR, suggesting sterile immunity in these mice. In line with previous studies [3], [40], [41], the protection efficacy correlates in both infection models with the neutralizing antibody titer, suggesting that the antibody-mediated inhibition of the initial virus infection might be the key factor for protection.

In conclusion, we demonstrated the efficacy of a combinatory DNA vaccine comprised of two codon-optimized expression plasmids to protect against two severe viral respiratory tract infections. A comprehensive analysis revealed evidence of immunological interference for the cellular, but not for the humoral response. Taken together, this combinatory vaccine against RSV and IAV could have great implications on the rate of severe respiratory tract infections and could further reduce the number of necessary vaccinations.

## Supporting Information

Figure S1 **HA-specific CD4^+^ T-cell responses.** Balb/c mice were immunized according to Table 1. Since the low dose regimen did not result in substantial responses, only the groups which received a total of 40 µg of plasmid DNA were included. A) IAV-HA-specific CD4^+^ T-cell responses were analyzed one week after the second immunization by intracellular staining for the inflammatory cytokines IFN-γ, TNF and IL-2. The percentages of the different populations among the total CD4^+^ T-cells are shown. Mean values and standard errors of the means (SEM) represent 8 mice per vaccine group out of two independent experiments and 4 mice for the naïve group. (*** = p<0.001, ** = p<0.01, * = p<0.05; 1 way-ANOVA, Tukey post-test). B) Splenocytes were re-stimulated for 48 h in the presence of the HA-specific peptide and anti-CD28 antibody. Supernatants were analyzed in cytokine-specific ELISA for IL-4, IL-5, IL-10 and IL-13 (eBioscience, Frankfurt, Germany). Since no production of IL-5 and IL-10 could be detected, only the results for IL-4 and IL-13 are shown. Mean values and standard error of the means (SEM) represent 4 mice per vaccine group. The control group received 20 µg of empty pcDNA and 20 µg of empty pVAX. (*** = p<0.001, ** = p<0.01, * = p<0.05; 1 way-ANOVA, Tukey post-test).
(PDF)
