# Supplementary figures and images for: Protective Efficacy and Immunogenicity of a Combinatory DNA Vaccine against Influenza A Virus and the Respiratory Syncytial Virus
Source: PLoS One. 2013 Aug 14;8(8):e72217. doi: 10.1371/journal.pone.0072217 (PMC3743785; doi:10.1371/journal.pone.0072217)

**A**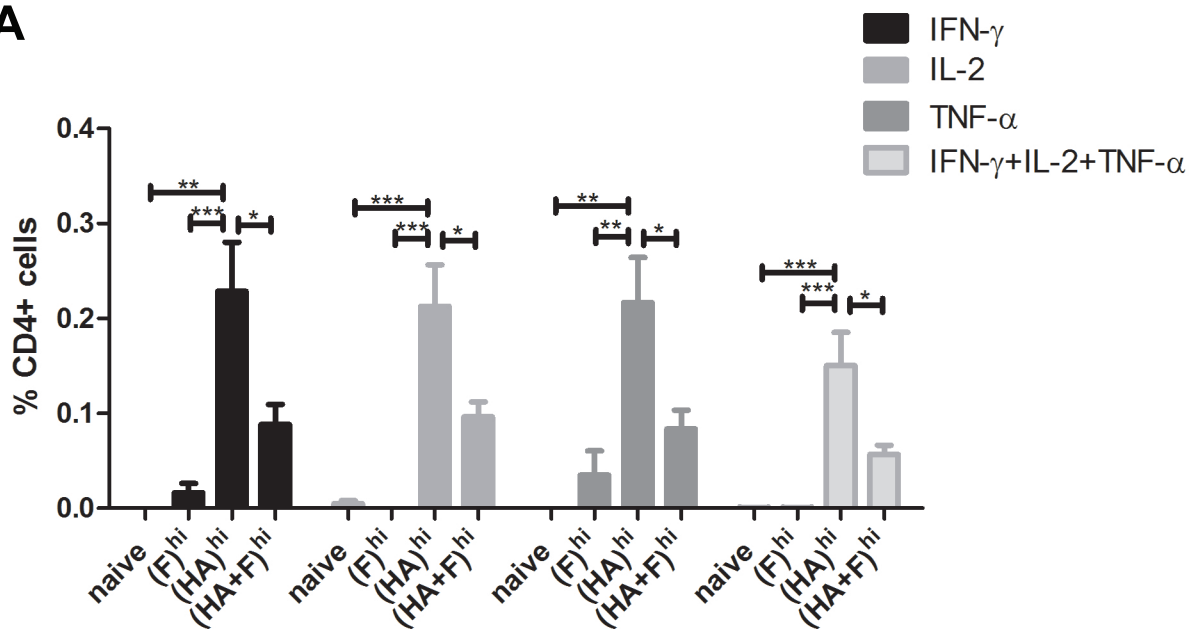**B**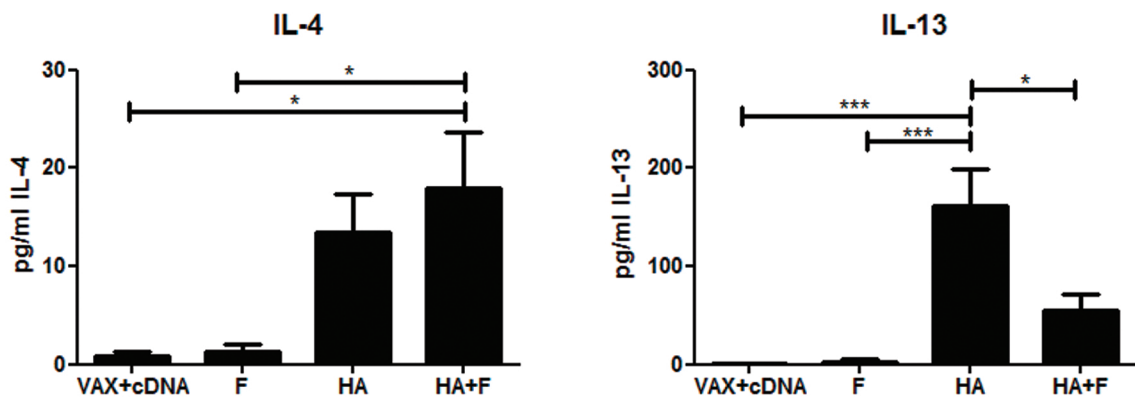

Supplement: Figure S1 — HA-specific CD4+ T-cell responses. Balb/c mice were immunized according to Table 1. Since the low dose regimen did not result in substantial responses, only the groups which received a total of 40 µg of plasmid DNA were included. A) IAV-HA-specific CD4+ T-cell responses were analyzed one week after the second immunization by intracellular staining for the inflammatory cytokines IFN-γ, TNF and IL-2. The percentages of the different populations among the total CD4+ T-cells are shown. Mean values and standard errors of the means (SEM) represent 8 mice per vaccine group out of two independent experiments and 4 mice for the naïve group. (*** = p<0.001, ** = p<0.01, * = p<0.05; 1 way-ANOVA, Tukey post-test). B) Splenocytes were re-stimulated for 48 h in the presence of the HA-specific peptide and anti-CD28 antibody. Supernatants were analyzed in cytokine-specific ELISA for IL-4, IL-5, IL-10 and IL-13 (eBioscience, Frankfurt, Germany). Since no production of IL-5 and IL-10 could be detected, only the results for IL-4 and IL-13 are shown. Mean values and standard error of the means (SEM) represent 4 mice per vaccine group. The control group received 20 µg of empty pcDNA and 20 µg of empty pVAX. (*** = p<0.001, ** = p<0.01, * = p<0.05; 1 way-ANOVA, Tukey post-test). (PDF) [file pone.0072217.s001.pdf]
